# Evaluation of Interactions of Selected Olivacine Derivatives with DNA and Topoisomerase II

**DOI:** 10.3390/ijms22168492

**Published:** 2021-08-06

**Authors:** Beata Tylińska, Agnieszka Dobosz, Jan Spychała, Łucja Cwynar-Zając, Żaneta Czyżnikowska, Amadeusz Kuźniarski, Tomasz Gębarowski

**Affiliations:** 1 Department of Organic Chemistry, Wroclaw Medical University, 50-556 Wroclaw, Poland; beata.tylinska@umed.wroc.pl; 2 Department of Basic Medical Sciences, Wroclaw Medical University, 50-556 Wroclaw, Poland; jan.spychala@umed.wroc.pl (J.S.); lucja.cwynar-zajac@umed.wroc.pl (Ł.C.-Z.); tomasz.gebarowski@umed.wroc.pl (T.G.); 3 Department of Inorganic Chemistry, Wroclaw Medical University, 50-556 Wroclaw, Poland; zaneta.czyznikowska@umed.wroc.pl; 4 Department of Prosthetic Dentistry, Wroclaw Medical University, 50-425 Wroclaw, Poland; amadeusz.kuzniarski@umed.wroc.pl

**Keywords:** comet assay, polarography, pyridocarbazoles, etoposide, olivacine derivatives, histone acetylation, molecular modelling

## Abstract

Olivacine and ellipticine are model anticancer drugs acting as topoisomerase II inhibitors. Here, we present investigations performed on four olivacine derivatives in light of their antitumor activity. The aim of this study was to identify the best antitumor compound among the four tested olivacine derivatives. The study was performed using CCRF/CEM and MCF-7 cell lines. Comet assay, polarography, inhibition of topoisomerase II activity, histone acetylation, and molecular docking studies were performed. Each tested compound displayed interaction with DNA and topoisomerase II, but did not cause histone acetylation. Compound **2** (9-methoxy-5,6-dimethyl-1-({[1-hydroxy-2-(hydroxymethyl)butan-2-yl]amino}methyl)-6*H*-pyrido[4,3-*b*]carbazole) was found to be the best candidate as an anticancer drug because it had the highest affinity for topoisomerase II and caused the least genotoxic damage in cells.

## 1. Introduction

Olivacine and ellipticine are alkaloids containing the pyrido[4,3-*b*]carbazole system, discovered in the mid-20th century [1]. They both show anticancer effects, but the mechanism of their action is still not explained precisely. Studies have shown the possibility of interaction with DNA. Ellipticine has the appropriate shape and size, similar to the purine and pyrimidine base pairs, to intercalate into the double DNA strand. Additionally, the polycyclic nature of this cytostatic allows it to penetrate the hydrophobic DNA regions [2]. As demonstrated in a crystallographic model, ellipticine interacts with DNA bases and is aligned parallel to the hydrogen bonds between the base pairs. The result is that DNA is lengthened and unwound [3].

The second mechanism of the anticancer action of olivacine and ellipticine is by acting as topoisomerase II inhibitors. Topoisomerase II binding by ellipticine increases the number of unrepaired DNA strand breaks. Such topoisomerase II-DNA-ellipticine complex formation leads to an increase in the amount of unrepaired DNA damage and consequently causes cell death. However, it is not known whether topoisomerase II is necessary in the initial stage of the drug’s action and whether the drug binds first with DNA or with topoisomerase and after that, a three-component complex is formed [4,5,6]. This is why the new ellipticine and olivacine derivatives should be studied in terms of their direct interactions with nucleic acid, the affinity to DNA, and their influence on electrochemical DNA reduction.

**Figure 1:**
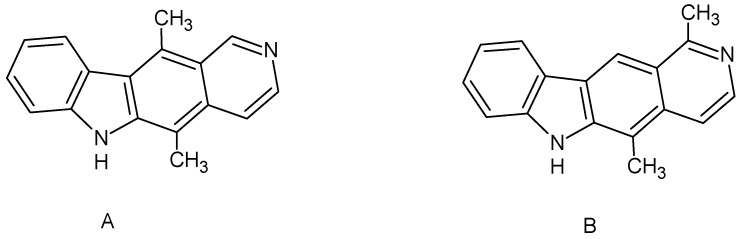
Chemical structures of (**A**) ellipticine (5,11-dimethyl-6*H*-pyrido[4,3-*b*]carbazole) and (**B**) olivacine (1,5-dimethyl-6*H*-pyrido[4,3-*b*]carbazole).

Ellipticine, a naturally occurring compound, can be isolated from Apocynaceae plants such as *Excavatia coccinea* or *Ochrosia borbonica*, and was isolated for the first time in 1959 from *Ochrosia elliptica* [7,8]. The alkaloid olivacine was first isolated a year before ellipticine from *Aspidosperma olivaceum* and three other plants: *Aspidosperma longipetiolatum*, *Aspidosperma australe*, and *Tabernaemontana psychotrifolia* [9]. The mentioned compounds are isomers of each other (Figure 1).

Since olivacine and ellipticine were revealed to be anticancer compounds, some attempts have been made to modify these compounds to increase their antitumor activity. One of the newly synthesized ellipticine derivatives was elliptinium. This compound was used in France for the treatment of metastatic breast cancer, but after several years of use, it was withdrawn due to adverse side effects (severe xerostomia and vomiting, hemolysis, phlebitis, fatigue, muscle cramps) [10]. The other interesting olivacine derivative was S16020, which reached phase II clinical investigations [11,12,13]. Unfortunately, side effects have so far been an obstacle in the use of these compounds for medical treatment [14]. However, attempts to obtain new anticancer ellipticine and olivacine derivatives are still being made [15,16].

**Figure 2:**
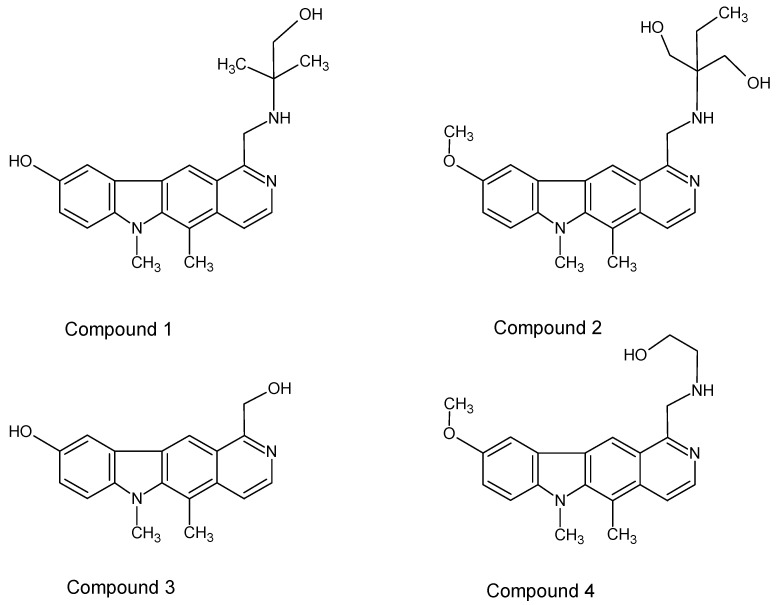
Derivatives of olivacine investigated in this study. **Compound 1:** 9-hydroxy-5,6-dimethyl-1-{[(1-hydroxy-2-methylpropan-2-yl)amino]methyl}-6*H*-pyrido[4,3-*b*]carbazole; **Compound 2:** 9-methoxy-5,6-dimethyl-1-({[1-hydroxy-2-(hydroxymethyl)butan-2-yl]amino} methyl)-6*H*-pyrido[4,3-*b*]carbazole; **Compound 3:** 9-hydroxy-5,6-dimethyl-1-hydroxymethyl-6*H* -pyrido[4,3-*b*]carbazole; **Compound 4:** 9-methoxy-5,6-dimethyl-1-{[(1-hydroxy-ethyl-2-yl)amino] methyl}-6*H*-pyrido[4,3-*b*]carbazole.

In this study, some new olivacine derivatives (Figure 2) were used in experiments as well as ellipticine itself (Figure 1), as a model compound. The aim of this study was to find out how they can interact with DNA to extend our knowledge about this process. In living cells, the interaction of chemical compounds with DNA can be analyzed using the single cell electrophoresis test, also called the comet assay. It allows for quantitative analysis of DNA breaks in cells exposed to the damaging agent. The comet assay is based on the ability of negatively charged fragments of DNA to migrate through the agarose gel in an electric field. The extent of DNA migration is connected directly with DNA damage inside the cells [17]. Measuring the amount of damage consists of analyzing the amount of DNA in the head and the tail of a comet as well as the length of the comet tail. When performing the test in alkaline conditions (pH 13.0), mostly single-strand and secondary breaks are detected. These breaks are formed in places sensitive to alkaline pH. The comet assay has never been used before to examine pyridocarbazole interactions with DNA.

The studied compounds (Figure 2) were previously tested by Jasztold-Howorko et al. [15] in relation to their anticancer activity, and by Gębarowski et al. [18] in order to find out how they influence the level of p53 protein. The paper of Gębarowski et al. [18] presented the clinical effect of pyridocarbazole action with DNA. In this study, we wanted to focus on the mechanism of the compounds’ antitumor action toward DNA using the comet assay and topoisomerase II, measuring the inhibition of topoisomerase II activity. Topoisomerases are required for proper DNA synthesis and gene transcription. Inhibition of topoisomerase II function leads to the formation of numerous intra-DNA strand bonds, which block basic life processes—transcription and replication. Strong expression of topoisomerase II is characteristic for tumor cells, and the use of a topoisomerase inhibitor such as ellipticine causes inhibitor-induced DNA damage, cell cycle arrest, and ultimately activation of apoptosis [19].

Analyzing the studies of new compounds affecting DNA, many questions arise regarding their mechanism of action. They concern both cases when DNA breaks are visible in the comet assay and when they are missing. This was the rationale for us to investigate the mechanism of interaction of the new pyridocarbazole derivatives with DNA. We also wanted to evaluate the usefulness of polarography to analyze the interactions of the new compounds with DNA.

Nucleic acids are electrochemically active, as was proven by Emil Paleček at the end of the 1950s [20], and undergo redox processes on various electrodes such as mercury or solid (carbon, gold, graphite). However, the whole nucleic acid molecule cannot undergo the redox process: sugar and phosphoric moieties are not polarographically active; only bases can undergo the process [21]. Adenine and cytosine are reduced on a mercury electrode, giving reduction peaks in the range of −1.4 V to −1.5 V in acidic or neutral aqueous solutions [21,22]. Under these conditions, guanine is reduced at much more negative potentials, which makes direct measurement impossible. Guanine and adenine are oxidized on carbon electrodes [23,24]. Due to this feature, it is possible to use electrochemical methods to study the interactions of nucleic acids with other compounds (drugs, metal ions) and to track changes in their structure related to these interactions. Alternating current (AC) polarography, differential pulse (DPP) polarography, and cyclic voltammetry (CV) are the most useful techniques in such studies. We used these methods in our study to explore the mechanism of action of newly synthesized pyridocarbazole derivatives with DNA and to complete and extend the results achieved during the comet assay. We wanted to deeply understand the mechanism of action of these anticancer compounds.

To further enhance our knowledge of how pyridocarbazoles influence DNA, we also focused on nucleosomal histone acetylation. Histones are basic proteins that are part of chromatin and may be chemically modified by enzymes to regulate gene transcription. The two main processes that modulate chromatin structure and function and/or gene expression in cells are acetylation and deacetylation of nucleosomal histones. There are two classes of enzymes that control the equilibrium of histone acetylation: histone acetyltransferases (HATs) and histone deacetylases (HDACs) [25]. The acetylation of lysine side chains by HATs neutralizes the lysine positive charge and consequently weakens DNA/histone interactions [26]. When there is an imbalance in the equilibrium between HATs and HDACs, it may cause cancer progression in cells. HDAC inhibitors (HDACi) are able to induce apoptosis in cancer cells and for this reason, demonstrate antitumor activity [27,28]. We wanted to find out if our compounds can act as HDAC inhibitors.

## 2. Results

### 2.1. Preliminary Research

**Figure 3:**
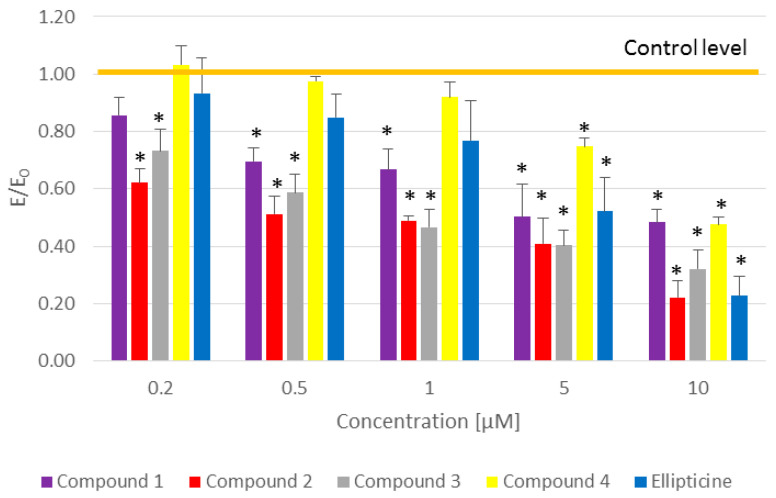
The MTT assay performed for the studied pyridocarbazoles and ellipticine. Concentration of all tested compounds was 0.2–10 µM. E/E_0_ is the mean value in reference to the control (E_0_ = control = 1); * *p* ≤0.05.

In the first step, our aim was to evaluate the cytotoxic effect of the tested compounds in the concentration range 1–10 µM in the image-based cytometer Arthur (NanoEnTek Inc.). After 2 h of the test, no significant increase in dead cells was observed. Additionally, the MTT assay was performed to assess cell viability in the standard time of 24 h. The results confirmed the high activity of the tested compounds, especially compound **2**. Concentration-dependent activity was observed in the CCRF/CEM cells. The results of this assay are shown in Figure 3.

### 2.2. Comet Assay

Due to the strong antitumor activity in the model studies including the relatively high activity of cells exhibiting multidrug resistance traits, many research centers have been trying to identify ellipticine and olivacine derivatives that exhibit less toxicity in vivo while retaining the antitumor efficacy of the parent formulations. In this study, we evaluated the antitumor effects of four olivacine derivatives (Figure 2).

The first stage of the study was to test the genotoxic effect of selected pyridocarbazole derivatives on DNA using the comet assay and to compare the results to those of ellipticine. A concentration ≤10 µM was selected for the comet assay due to the cytotoxicity of the compounds to cells. In our experiment, the acute human lymphoblastic leukemia cell line CCRF/CEM was used.

**Figure 4:**
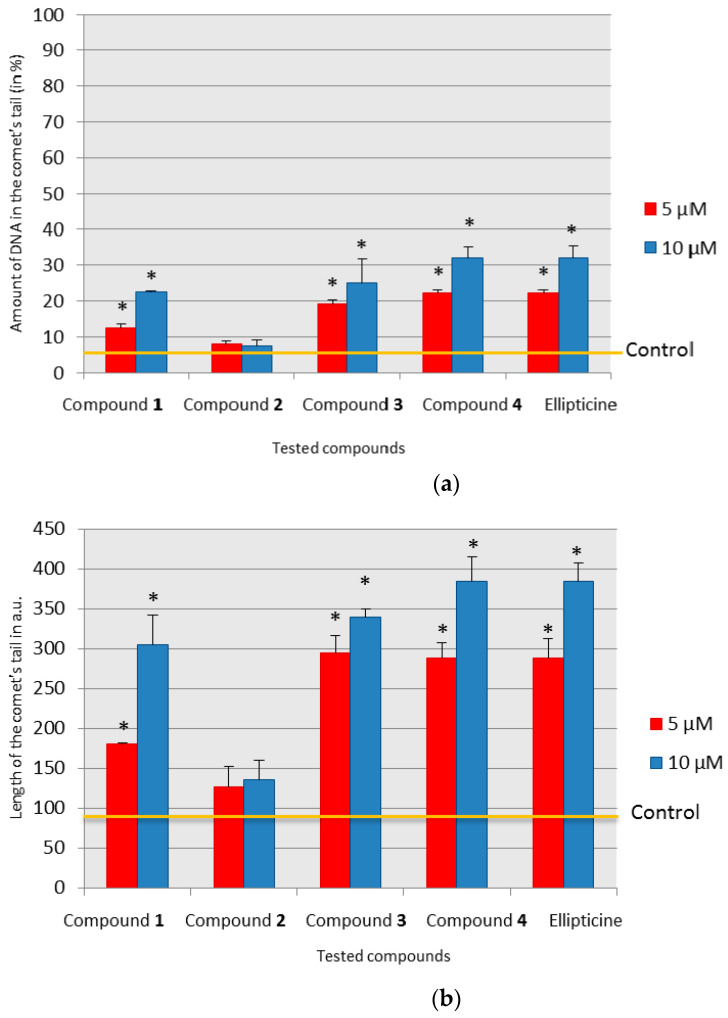
Evaluation of direct genotoxicity in a comet test; * *p* ≤0.05. (**a**) The figure shows the amount of DNA in the comet’s tail (in %). Control value indicated by yellow line: 5.42 (±1.33). (**b**) The figure shows the length of the comet’s tail in arbitrary units (a.u.). Control value indicated by yellow line: 88.2 (±4.6).

The comet assay is a very good and sensitive technique to detect chromosomal DNA damage in cells. The aim of this assay was to check the genotoxic action caused by the tested compounds under the conditions of inhibition or minimization of the repair processes. The cells were exposed to our four pyridocarbazole compounds and to ellipticine, and this genotoxic action can be seen in the form of DNA damage (Figure 4a,b).

The percentage of DNA in the tail of the comet was chosen to assess the amount of caused damage. It is a linear parameter that shows the extent of damage in DNA and is recommended by many authors [29,30]. The amount of DNA in comet tails was compared to the control value using the *t*-test.

In the comet assay, ellipticine and compound **4** showed the strongest genotoxic effects. The least genotoxic damage in the form of DNA chain fractures was observed in the comet assay for compound **2**. At both concentrations, compound **2** showed no significant differences from the control (Figure 4a).

In addition, the second linear parameter—the length of the comet’s tail—was chosen to assess the genotoxic effect of the compounds. This parameter also confirmed the strongest genotoxic effect of compound **4** and ellipticine and the absence of a significant, compared to the control, genotoxic effect of compound **2**. In the case of the remaining tested compounds, we observed an increase in the amount of genotoxic damage according to the concentration. After performing the *t*-test, a statistically significant amount of damage was already visible for compound **4** at 5 µM, ellipticine and compound **1** at 10 µM, and compound **3** at 5 µM. In the case of compound **1** and at the concentration of 5 µM, the tail length was almost 40% shorter compared to that of compounds **3** and **4** and ellipticine (Figure 4b).

### 2.3. Electrochemical Investigations

The comet assay shows the effects of substances interacting with DNA in cells. Different measurement results have shown that the tested compounds damage DNA in different ways and with different degrees. Among all tested compounds, compound **2** had the weakest genotoxic effect. We have tried to explain the mechanism of interactions with DNA by means of polarography to determine how it can interact with DNA.

Electrochemical investigations were performed using acetate buffer (pH 5.5) as the pouring medium suitable to measure DNA reduction at the hanging mercury drop electrode (HMDE). As described before, only adenine and cytosine were reduced on the mercury drop electrode in aqueous solutions. However, this process must be preceded by protonation of the bases—N(1) of adenine and N(3) of cytosine atoms [31,32]. This is the reason why acidic buffer must be used for electrochemistry to provide suitable conditions. Moreover, in the electrochemical studies, neither Na^+^ nor CH_3_COO^−^ ions significantly affect the conformational changes in the native DNA structure.

**Figure 5:**
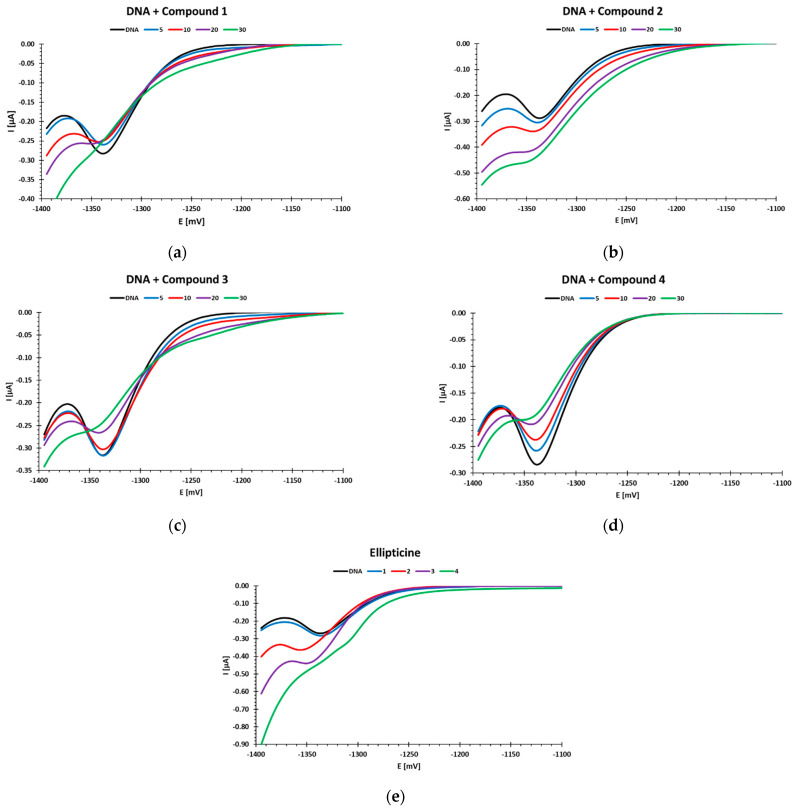
Polarographic curves showing the dependence of the DNA reduction peak on the concentration of the tested compounds: (**a**) compound **1**; (**b**) compound **2**; (**c**) compound **3**; (**d**) compound **4**; (**e**) ellipticine. Concentration of compounds **1–4** varied from 5–30 µM and ellipticine from 1–4 µM and is shown in legend of each subfigure in µM, DNA concentration was constant (25 µg/mL).

Polarographic measurements have shown that the tested compounds interact with DNA in different ways. Three of them caused a decrease in the DNA reduction peak with a potential of about −1.35 V (compounds **1**, **3** and **4**), and two of them (compound **2** and ellipticine) increased the DNA reduction peak (Figure 5). In the case of ellipticine, the changes are quite rapid and cause the loss of structure of DNA, already visible in polarographic measurements when the concentration 4 µM is applied.

Moreover, in the case of compounds **1** and **3**, there was an additional DNA reduction peak with a potential of about −1.2 V (Figure 5a,c). This peak indicates the presence of short, double-stranded DNA fragments in the test sample. Previous research performed by our team has shown that such a polarographic result was obtained during the measurement of sonicated DNA. DNA sonication leads to cutting of the native acid structure and the formation of short, double-stranded fragments [33].

The decrease in the DNA reduction peak may be caused by the binding of cytostatic to nucleic bases. This increases the stability of DNA chains at lower concentrations and reduces the amount of bases available for reduction. The use of higher concentrations of compounds leads to destabilization of the DNA structure. The polarographic image of this process is the disappearance of the DNA reduction peak with a potential of approximately −1.35 V. It can be seen in the case of compounds **1**, **3**, and **4** (Figure 5a,c,d). Destabilization of the DNA structure when increasing the concentration of compounds excludes binding of the compounds to the phosphate residues. Binding of phosphate residues in the polynucleotide chain weakens the adverse effects of negatively charged fragments between themselves and the negatively charged electrode (repulsion). This leads to increased efficiency of hydrogen bonds and increased stability of the native DNA structure [34]. In addition, a relatively high concentration of Na^+^ ions (0.2 M acetate buffer) stabilizes the DNA native structure by binding them to the phosphate residues, while making it difficult for negatively charged phosphate residues to interact with other compounds. The destabilization of the DNA native structure observed in polarographic measurements under the influence of higher concentrations of the examined cytostatic agents indicates the mechanism of intercalation of these compounds into the DNA chain. The resulting DNA damage in a cell under the influence of compounds **1**, **3**, and **4** was visible in the comet test (Figure 4a,b). The differentiation of the dynamics of formation and the amount of damage visible in this test was also visible in polarography.

The most important parameter evaluated by polarography is the increase and decrease of the reduction peak. Table 1 shows the electrochemical parameters of the reduction peaks as well as the ratio of the current of the reduction peak formed after mixing the compounds with DNA (I_p_) to the current measured for DNA alone (I_pDNA_). A decrease was observed in the DNA reduction peak compared to the control, which is presented as I_p_/I_pDNA_ = 1 (for DNA without any compound). Table 1 shows that compound **1** caused the smallest decrease in the reduction peak (0.92 compared to control), but the fastest of all compounds caused DNA destabilization when its concentration was 20 µM. Compounds **3** and **4** reduced the reduction peak by 0.84 and 0.79, respectively, compared to the control and destabilized DNA at the concentration of 30 µM.

In the case of compound **2** and ellipticine, an increase in the reduction current could be observed. Such a situation, when the increase in the DNA current reduction peak is followed by an increase in the concentration of these compounds, is evidence of double helix destabilization [33,34]. The mechanism of this process is probably the same as for the other investigated derivatives (intercalation). The observed increase in the DNA current reduction peak may be caused by the binding of ellipticine and compound **2** to the bases that do not undergo the reduction process on the mercury drop electrode such as thymine and guanine. Hence, more bases could reduce themselves on the mercury drop electrode during polarographic measurement, and this was the reason why the reduction peak increased [22]. This interaction of compound **2** with DNA cannot be observed in the comet assay and only revealed some not significant genotoxic damage when compared to the control (Figure 4a,b).

**Figure 6:**
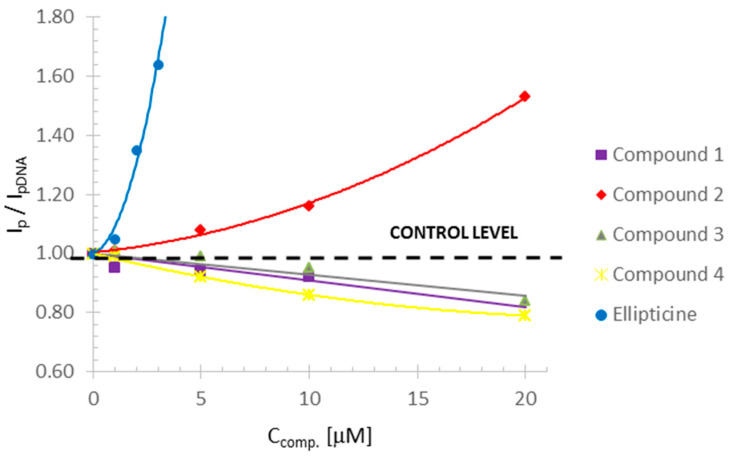
Effect of tested compound concentration on DNA reduction current compared to the control (K = 1). Voltage values of the current according to Table 1.

Changes for ellipticine observed in polarography begin earlier than for compound **2.** It can be seen in Table 1 that the changes started at the concentration of 1 µM, significant DNA destabilization appeared at 2 µM, and total destabilization at 4 µM. This is consistent with the comet assay, where genotoxic damage can be seen at similar concentrations. Both ellipticine and compound **2** can damage DNA structure at higher concentrations. The positive slope of the simple regressions for compound **2** and ellipticine may indicate a similar mechanism of interaction of these compounds with DNA. On the other hand, the negative slope of the simple regressions of compounds **1**, **3**, and **4** may confirm their similar interaction with DNA, different from compound **2** and ellipticine (Figure 6).

It is significant that despite the different mechanisms of the compounds’ interactions with DNA, the final effect of their high concentrations was similar. The differences are the result of the mechanism that leads to this process. This fact is not visible when performing the comet assay. In the comet assay, there are also no similarities between the mechanism of action of compound **2** and ellipticine, which were observed in the polarographic measurements (Figure 5 and Figure 6).

### 2.4. Inhibition of Topoisomerase II Activity

The important mechanism for the antitumor effects of ellipticine [4,5,6,35], olivacine, and their derivatives [12,35,36] is the inhibition of topoisomerase II (topo II) activity. To confirm whether the tested olivacine derivatives also interact with topo II, a series of comparative studies were performed. First, we wanted to compare the genotoxicity of a standard topoisomerase II inhibitor named etoposide (ETP) [37] and our four selected pyridocarbazole derivatives. It has been proven that etoposide reduces the activity of topoisomerase IIα in clinical use [38]. We decided to evaluate changes in the amount of DNA damage due to different combinations of topoisomerase II inhibitor and the tested compounds. First, we defined the concentration of topoisomerase II inhibitor causing a constant amount of DNA damage, and second, we evaluated the changes in the amount of genotoxic damage due to the different sequences of addition of the topoisomerase II inhibitor and the tested compounds.

The investigations were performed using the CCRF/CEM cell line. The cells were incubated with different concentrations of etoposide (2.5 µM, 5 µM, 10 µM, and 20 µM) for 20 or 60 min in the dark, at 4 °C. Then, the samples were tested using the comet assay. Based on the obtained results, the range of etoposide concentrations in which the constant amount of DNA damage in the cells was present and the optimal incubation time of CCRF/CEM cells with a standard cytostatic were determined. To determine the amount of damage, the percent of DNA in the tail was chosen. This parameter is often used to assess the degree of DNA relaxation caused by induced breaks in nucleic acid strands [11,37]. Topoisomerase II inhibitors such as etoposide were investigated by Salti et al. using the comet assay to detect DNA cleavages in cultured cells [39].

The amount of DNA damage caused under specific laboratory conditions and time is necessary to determine the concentration of etoposide in further comparisons with the tested pyridocarbazoles. During the incubation, the level of the damage was observed both in low and high etoposide concentrations. In the low etoposide concentration, the gradually increasing formation of topoisomerase II complexes was observed, while in the high etoposide concentration, strand breaks were observed. The experiment showed that the most effective etoposide concentration was 7.5 µM (as the average value in the range between 5 µM and 10 µM, where a constant amount of DNA damage in the cells was present), and the most efficacious incubation time was 20 min.

The aim of the study was to determine whether there was any correlation between the sequence of serving the etoposide and pyridocarbazole derivatives and the amount of genotoxic damage in DNA. We wanted to reveal whether the investigated pyridocarbazoles, similar to ETP, form cleavable complexes with topoisomerase II and DNA, which induce strand breaks, and how the earlier application of etoposide will affect the amount of damage and the subsequent administration of selected tested compounds; and vice versa, whether the use of tested derivatives in the beginning would affect the genotoxic properties of ETP. Only two compounds were chosen for the comet assay: compound **2** with the weakest genotoxic effect and compound **4** with the strongest genotoxic effect, which was proven using the comet assay (Figure 4a).

The results were compared to the damage caused by ETP alone and were calculated on the basis of three different tests: comet tail length, DNA content in the tail [40], and olive tail moment (OTM) (Table 2). OTM is a parameter useful in describing heterogeneity of a cell population, because it can collect variations in DNA distribution within the tail [41].

The genotoxic damage caused by etoposide itself in each test was set as the reference value of 100% (marked as 1 for each test). Because we performed three different tests, the reference value for etoposide was set as E_ETP_ = 3. Then, the same three experiments were performed for compounds **2** and **4**, and the amount of the genotoxic damage was established for each experiment. Next, these values were compared to our reference value for etoposide (E _ETP_ = 3), summed up, and are presented in Table 2.

When pyridocarbazoles were added to CCRF/CEM cells before etoposide, genotoxic damage was restricted to the range 1.53–1.71 for compound **2** and 2.51–2.82 for compound **4**, in comparison to the value E_ETP_ = 3, achieved by etoposide. Since the genotoxic effect of ETP is mainly related to the formation of topo II and DNA complexes, the decrease in the amount of damage may indicate either a direct effect of compounds and etoposide or competition between pyridocarbazole and ETP for access to DNA and topo II. This second mechanism seems to be more likely. The results indicate correlations between the amount of damage and the concentration of compounds (Table 2).

In the second case, when etoposide was added to CCRF/CEM cells before pyridocarbazoles, a slight increase of damage in relation to etoposide (3.06–3.19 for compound **2** and 3.12–3.32 for compound **4**) was observed. This means that this subsequent addition of pyridocarbazoles had no visible effect on the increase in DNA damage in the presence of compound **4**, although in direct interactions with DNA, compound **4** had the strongest genotoxic properties among all tested pyridocarbazoles. This indicates that in order to reveal its genotoxic properties, compound **4** must also form a complex of **4**-topo II-DNA, and the formation of such a complex is effectively blocked by the earlier use of ETP. These results indicate no correlations between the amount of damage and the concentration of the compounds (Table 2).

The next step of our investigations was to compare the genotoxic effect of compounds **2** and **4** to the sum of the damaging effect exerted together by etoposide and each compound (Table 3). As before, three different experiments were performed: comet tail length, DNA content in the tail, and olive tail moment (OTM) were measured. These three experiments were performed for compounds **2** and **4** at different compound concentrations (5, 10, and 20 µM) and for etoposide at the concentration of 7.5 µM. The amount of genotoxic damage was calculated for ETP itself and for each compound (**2** or **4**) separately. Then, the amount of genotoxic damage measured for ETP and for pyridocarbazoles was summed up mathematically at the level of 100% of damage. This 100% was established as the reference value, equal to 1 in every experiment. Because of the three different tests, the reference value for the sum of etoposide and pyridocarbazoles was indicated as E_ETP+PYR_ = 3. Once again, the experiments were performed twice: when ETP was added before and after the tested compounds. The values were compared to our reference value for the sum of etoposide and pyridocarbazoles (E_ETP+PYR_ = 3), and are presented in Table 3.

Table 3 presents the visible decrease in the amount of genotoxic damage when the tested pyridocarbazoles were used first, and then ETP was added to the system. Since the genotoxic effect of ETP is mainly related to the formation of topo II and DNA complexes, the decrease in the amount of damage may indicate either a direct effect of compounds and etoposide or competition between pyridocarbazole and ETP for access to DNA and the topo II complex. The results indicate correlations between the amount of damage and the concentration of the compounds.

In the case of earlier use of ETP before adding pyridocarbazole, a slight increase in the genotoxic damage was observed. It seems that subsequent addition of pyridocarbazoles to ETP has no visible effect on the increase in DNA damage in the presence of compound **4**, although in direct interactions with DNA, compound **4** showed genotoxic properties. This compound, in order to reveal its genotoxic properties, must also create a compound **4**-topo II-DNA complex, and the creation of such a complex effectively blocks the earlier application of ETP. The results allow us to conclude that, regardless of their effects on DNA, the tested derivatives competed for the same binding sites to the DNA-topo II complex as ETP itself. This confirms that one of the main mechanisms of action of our studied compounds was the inhibition of topoisomerase II activity, which in the case of other studied pyridocarbazoles has been reported earlier in the literature [4,5,6,35].

### 2.5. Histone Acetylation

**Figure 7:**
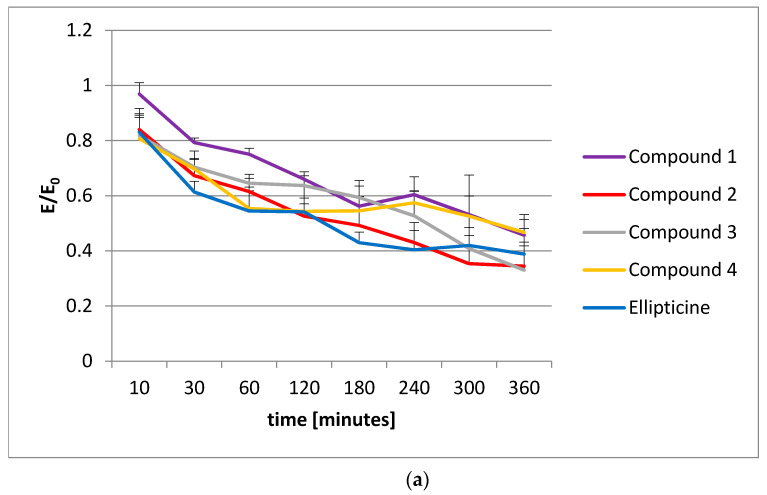

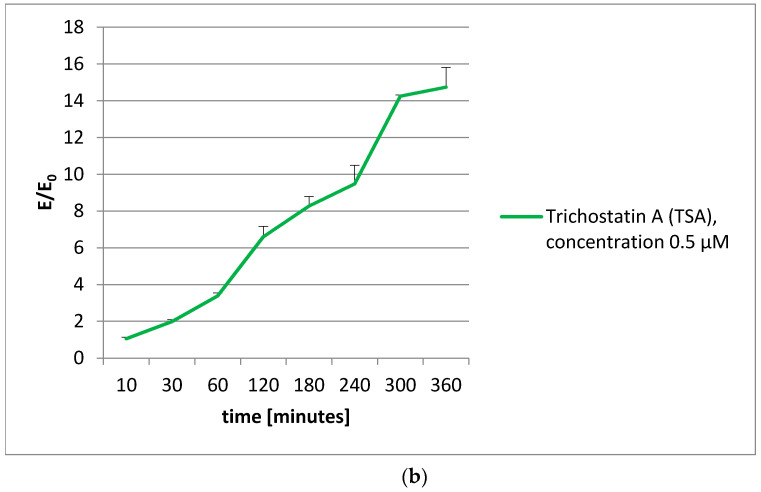
Histone acetylation, *n* = 5: (**a**) Studied compounds **1**–**4** and ellipticine in concentration of 10 µM; (**b**) trichostatin A (TSA) acetylation. E/E_0_ is the mean value in reference to the control (E_0_ = control = 1).

The last part of our study was performed to find out whether the studied pyridocarbazoles could induce histone acetylation. The experiment was performed for all studied compounds (**1**, **2**, **3**, **4**, and ellipticine) using the CycLex Cellular Histone Acetylation Assay Kit to evaluate the degree of acetylation caused by these compounds. The results are shown in Figure 7. The investigations were performed using the MCF-7 cell line.

The experiment showed that there was a decrease in the quantity of acetylated histones according to the time from the beginning of the experiment. This decrease was rather slow, but could indicate that the investigated pyridocarbazoles can cause histone acetylation. The results were compared to those obtained for the model histone acetylation inhibitor (HDAC) trichostatin A (Figure 7b), where significant histone acetylation can be observed.

**Figure 8:**
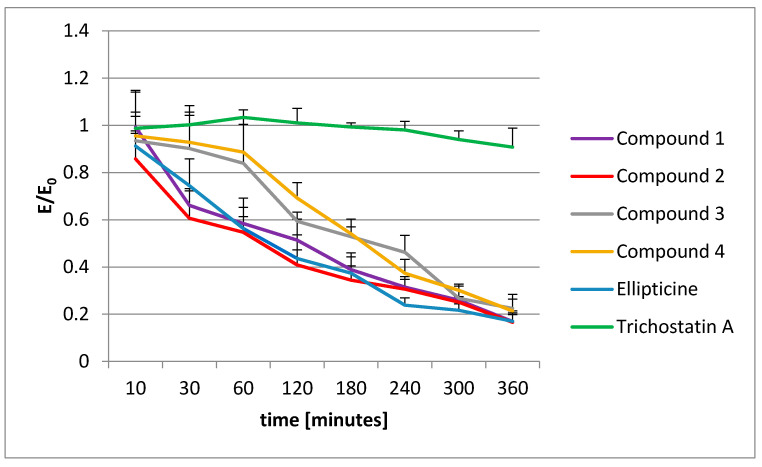
The MTT assay performed for studied pyridocarbazoles (compounds **1**–**4**), ellipticine, and trichostatin A. Concentration of all tested compounds was 10 µM. E/E_0_ is the mean value in reference to the control (E_0_ = control = 1).

To make sure that the observed histone acetylation was not significant and the tested compounds performed no histone acetylation, the results were compared with the MTT assay (Figure 8). The MTT assay shows the viability of cells and is widely used to measure the cytotoxic effects of different drugs on cell lines. It is based on the conversion of 3-[4,5-dimethylthiazol-2-yl]-2,5 diphenyl tetrazolium bromide (MTT) into formazan and is connected with the mitochondrial activity of the living cells (the higher the mitochondrial activity, the greater the cell viability) [42].

The tested compounds and ellipticine were incubated with CCRF/CEM cells and then observed in the MTT assay to see if there was an increase in the mitochondrial activity during that period. The results were compared with the results for the HDAC inhibitor trichostatin A, which causes no decrease in cell viability (Figure 8). Compounds **1**–**4** and ellipticine caused a decrease in the cell viability in that period (see Figure 8), which is additional, indirect proof that they do not induce histone acetylation.

The MTT assay indicated that the decrease of histone acetylation is connected with a decrease in the number of living cells, but not with the enzymatic activity of acetylase.

### 2.6. Molecular Modeling

**Figure 9:**
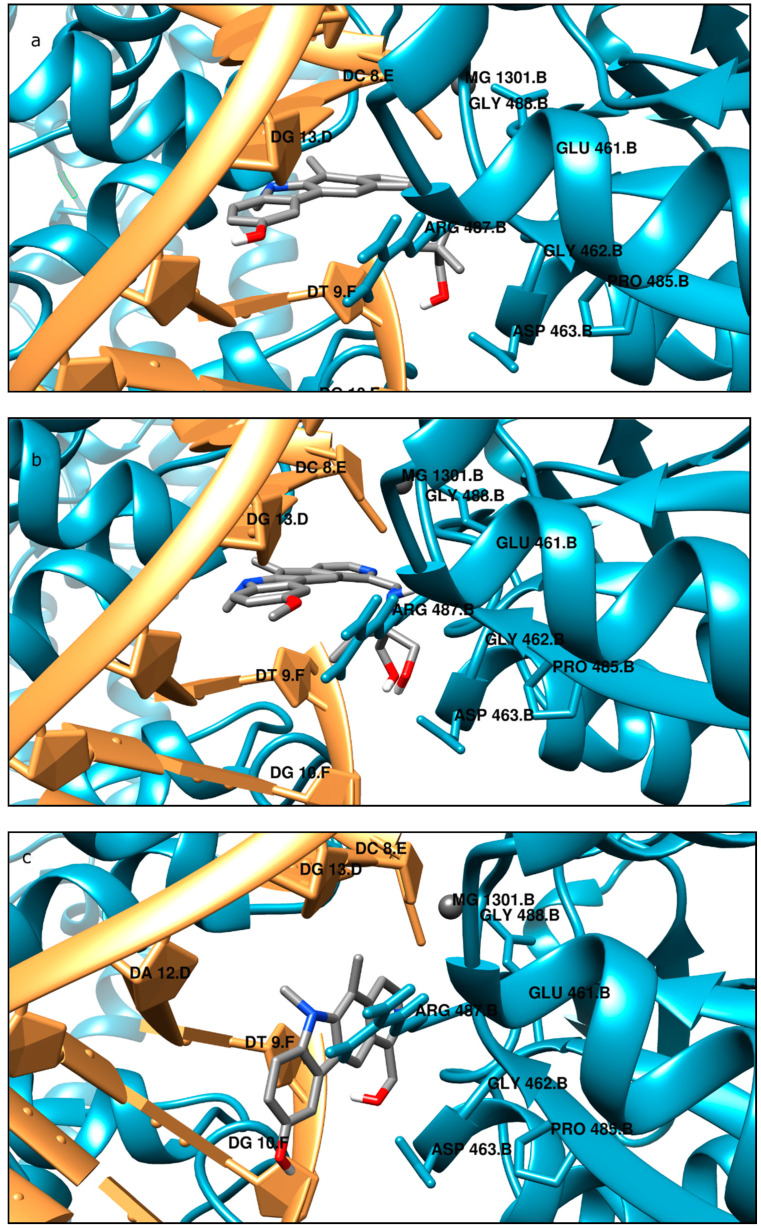

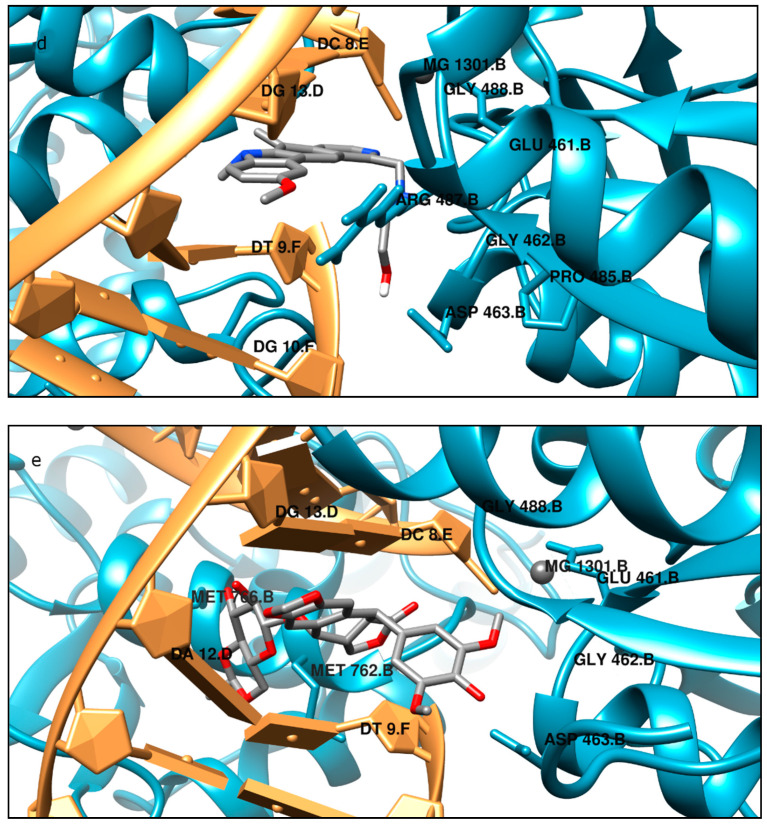
Binding mode of: (**a**) compound **1**; (**b**) compound **2**; (**c**) compound **3**; (**d**) compound **4**; (**e**) etoposide in the active site of topo IIα; 3D representation; DNA structure is colored gold, the protein chain is blue, ligand structures are grey.

**Figure 10:**
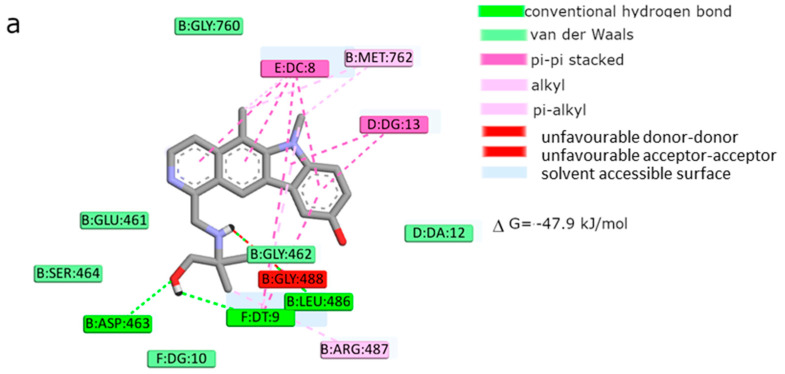

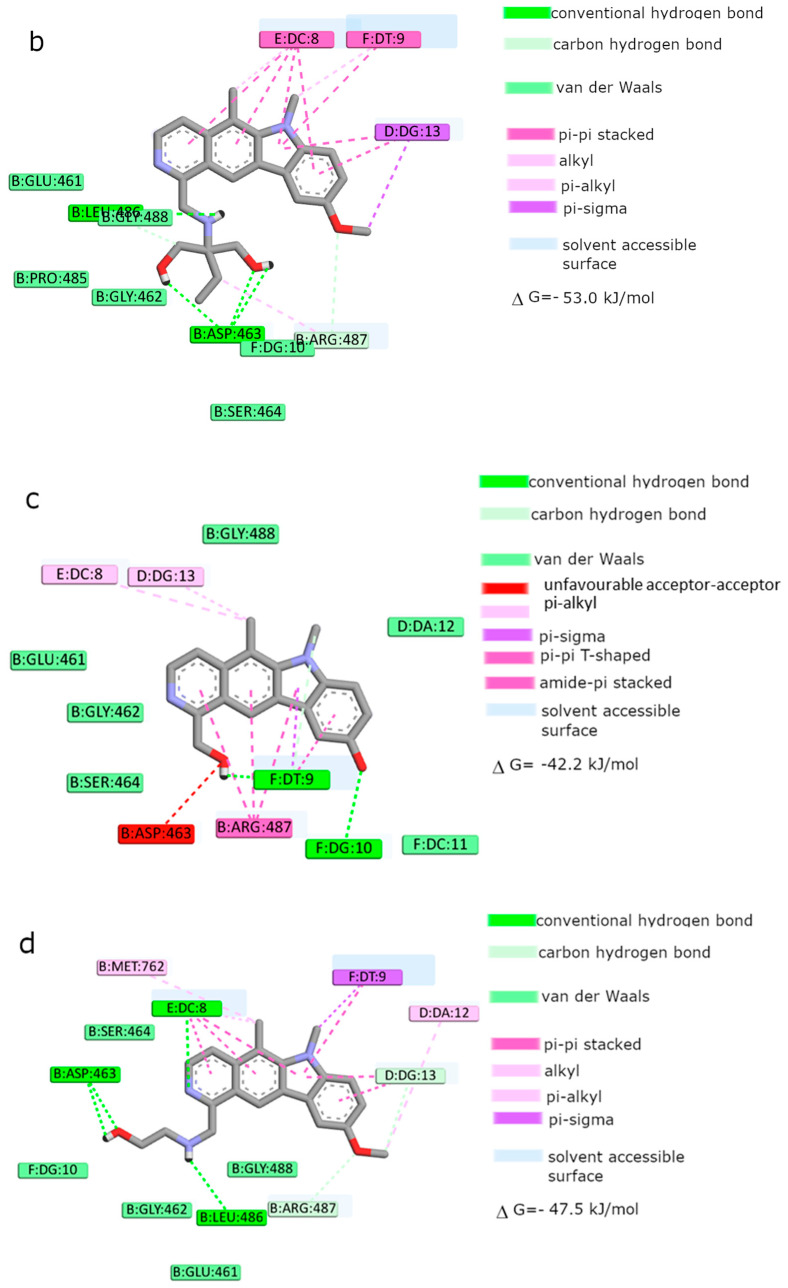

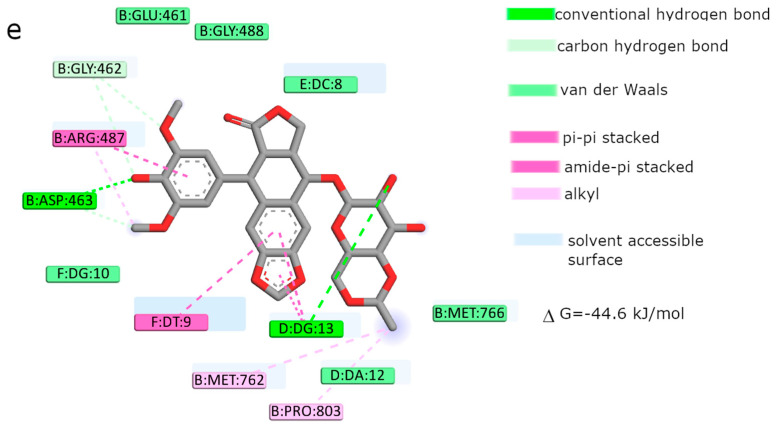
Free energy of binding and intermolecular interactions in the active site of topo IIα (2D representation) of: (**a**) compound **1**; (**b**) compound **2**; (**c**) compound **3**; (**d**) compound **4**; (**e**) etoposide.

In order to gain an insight into the binding mode of the analyzed compounds to the DNA and binding site of topo IIα, molecular modeling was performed. Taking into account the lowest scoring function energies, we analyzed in detail only the most possible binding modes. Validation of the docking procedure was performed by docking etoposide into the crystal structures of topoisomerase and comparing its position in the original crystallographic structure. Our results obtained for etoposide were consistent with previous studies [43,44]. As shown in Figure 9 and Figure 10, the aglycone part of etoposide is involved in π–π drug–DNA interactions with thymine DT9 and guanine DG13 rings. The analysis revealed the formation of conventional and carbon hydrogen bonds between guanine DG13, amino acids Gly462 and Asp463, and the inhibitor. The podophyllotoxin group of etoposide binds to the pocket surrounded by Glu461, Gly462, Asp463, Arg487, and Gly488 and is mainly responsible for drug–protein interactions. The suggested binding mode showed a free energy of binding value of −44 kJ/mol. According to the results the most potent topoisomerase ligand is compound **2** (ΔG_binding_ = −53 kJ/mol). The best docking pose showed that the pyridocarbazole moiety is involved in aromatic stacking interactions with DC8, DT9, and DG13 nucleotide residues. Additionally, the aliphatic chain of the inhibitor can form three hydrogen bonds between –OH groups and Asp436 amino acid residues. Next, the hydrogen bond between the –NH group and Leu486 is created. The complex is stabilized by van der Waals interactions with Glu461, Gly462, Ser464, Pro485, Gly488, and guanine DG10. Compound **4** showed a similar way of binding, but is a weaker inhibitor of topoisomerase, taking into account the estimated free energy of binding (see Figure 10). For example, the aliphatic chain of the inhibitor is involved in hydrogen bond creation between –OH and –NH groups and the amino acids Asp463 and Leu486. This part of the molecule interacts with Glu461, Gly462, Ser464, Gly488, and guanine DG10 via van der Waals interactions. The pyridocarbazole ring is directly exposed to π–π interactions with cytosine DC8, thymine DT9, and guanine DG13. One extra π–σ interaction with DT9 was observed. Alkyl interactions were also identified between substituents of pyridocarbazole and Met726 and adenine. The best docking pose of compound **1** revealed additional unfavorable donor–donor and acceptor–acceptor interactions with Gly488. Another origin of stabilization is π–stacking interactions between cytosine DC8 and guanine DG13 and pyridocarbazole rings. Three hydrogen bonds are also created between the aliphatic part of the inhibitor and thymine DT9, Asp463, and Leu486. The cavity created by Glu461, Gly462, Ser464, and Gly760 is responsible for van der Waals interactions. The binding manner of compound **3** is slightly different. Due to the smaller size and the shortening of the aliphatic chain, compound **3** interacts with DNA through van der Waals interactions and hydrogen bond interactions (see Figure 10). The cytosine DC8 and guanine DG13 bind via π–alkyl interactions. The position of the pyridocarbazole rings is mainly stabilized by π–π T-shaped, π–σ interactions, and amide–π stacked interactions with thymine DT9 and Arg487, respectively. Taking into account, the estimated free energy of binding (ΔG_binding_ = −42 kJ/mol), this compound may be a less effective topoisomerase inhibitor.

A summary is presented in Table 4.

## 3. Discussion

The anti-cancer properties of ellipticine and olivacine were discovered in the 1960s [45,46]. One of the ellipticine derivatives (ellipticine acetomethylate) was introduced for oncology treatment as celiptium; however, after several years of use, the compound was withdrawn due to multiple adverse side effects [47,48]. In turn, clinical trials of olivacine were stopped because the ratio of benefits to side effects was unsatisfactory [11]. However, many research teams continue studies on ellipticine and olivacine derivatives, which will exhibit less toxicity in vivo while retaining the anticancer efficacy of the parental compounds. This is also the reason why we have investigated toxicity and anticancer activity of four olivacine derivatives in this study.

Many studies have proven that olivacine, ellipticine, and their derivatives can interact with DNA and those interactions are omni-directional [49,50]. They interact with DNA by various mechanisms, the main ones being intercalation with DNA and inhibition of topoisomerase II activity [4,5,6]. Conditions conducive to the intercalation of ellipticine into double stranded DNA are provided by the size and shape of this compound, similar to purine and pyrimidine pairs, and in addition, the polycyclic nature of the cytostatic allows it to penetrate into the hydrophobic DNA regions [2,38]. Ellipticine also acts by topoisomerase II binding, leading to an increase in the number of unrepaired DNA strand breaks. The formation of the topoisomerase II-DNA-ellipticine complex leads to an increase in the amount of unrepaired DNA damage and ultimately to cell death. However, it is not known whether topoisomerase II is required for the initial action of the drug and whether the drug binds first to DNA or first to topoisomerase and then forms a ternary complex [4,5,6]. Therefore, new ellipticine and olivacine derivatives should be studied in terms of direct interactions with nucleic acid, the affinity of these compounds for DNA and their effect on electrochemical reduction of DNA bases. It was also reported by Zencir et al. that ellipticine and its derivatives can suppress telomere lengthening in cells, which can help to kill tumor cells [51].

Previous research has shown that the pyridocarbazoles studied here have anticancer activities [15,18]. A couple of investigations were performed to find low toxic, selective compounds for normal cells. We started to study the action of the new olivacine derivatives by testing their genotoxic effects in a comet assay and comparing them to ellipticine itself. These investigations are quite new, because we found only one paper in the literature describing the genotoxic effects of ellipticine studied in a comet assay on Chinese hamster cells: the CHO (hamster ovary cells) and DC3F (lung fibroblast cells) lines [19].

Among tested olivacine derivatives, compounds **1**, **3**, **4**, and ellipticine showed genotoxic effects, the strongest was for compound **4** and ellipticine. A statistically significant increase in the amount of damage, calculated by the *t*-test, in the case of compounds **3** and **4**, was observed at the concentration of 5 µM, and for ellipticine and compound **1** at the concentration of 10 µM. In the case of compound **2**, we found no genotoxic damage. To ascertain whether and how compound **2** can interact with DNA, we used the polarographic method.

For electrochemical studies, the acetate buffer (pH 5.5) was used, because it is suitable for analyzing the DNA reduction process at the mercury electrode. It is known that on a mercury drop electrode, in aqueous solutions, only adenine and cytosine are reduced. However, to occur, this reduction must be preceded by the protonation of bases—N(1) adenine atom and N(3) cytosine atom [31,32].

Three of the tested compounds (**1**, **3**, and **4**) caused a decrease in the DNA reduction peak at a potential of ca. −1.35 V, while two of them caused a peak increase. Moreover, in the case of compounds **1** and **3**, an additional DNA reduction peak appeared at a potential of ca. −1.2 V. This peak indicates the presence of short, double-stranded DNA fragments in the tested samples. Previous studies by our team have shown that such a polarographic result was obtained when measuring sonicated DNA [33]. DNA sonication leads to cutting of the native acid structure and the formation of short, double-stranded fragments [33].

The decrease in the DNA reduction peak may be caused by the binding of compounds to the nucleic bases. At lower compound concentrations, it caused an increase in the stability of DNA chains and a decrease in the number of bases available for reduction. The use of higher concentrations of the tested compounds led to destabilization of the DNA structure. Destabilization of the DNA structure with increasing concentrations of the tested compounds precluded binding of the compounds to the phosphate residues. The binding of phosphate residues in the polynucleotide chain attenuates the negative interactions of the negatively charged fragments with each other as well as with the negatively charged electrode (repulsion). This leads to increased hydrogen bonding efficiency and increased stability of the native DNA structure [20,34]. In addition, the relatively high concentration of Na^+^ ions (0.2 M acetate buffer) stabilized the native DNA structure by binding to the phosphate residues, while hindering the availability of negatively charged phosphate residues to interact with other compounds. At higher concentrations of the tested compounds, in the polarographic measurements, destabilization of the native DNA structure was observed, which may indicate intercalation of the tested compounds into the DNA chain. Similar DNA structural destabilization under the influence of compounds **1**, **3**, and **4** was also seen in the comet assay.

The tested compounds differed in the concentrations leading to the greatest amount of DNA damage. Compound **1** caused a slight decrease in the reduction peak to 0.92 compared to the control, and the DNA destabilization effect was evident at a concentration of 20 µM. Compounds **3** and **4** reduced the reduction peak to 0.84 and 0.79 (control = 1), respectively, and destabilized the DNA at a concentration of 30 µM.

An increase in the reduction peak in the polarographic studies was observed for compound **2** and ellipticine. The increase in the DNA reduction peak with the increase in the concentration of these compounds may indicate the destabilization of the double DNA helix [33,34]. The mechanism of this process was probably identical to the other derivatives tested (intercalation) [38,50]. This could have been caused by the binding of ellipticine and compound **2** to the nucleic bases that do not undergo reduction at the mercury drop electrode (thymine, guanine). Hence, it may have caused the observed higher amount of reducing bases on the mercury drop during the polarographic measurement and the increase in the reduction peak [20].

In the case of compound **2**, the polarographic results were not confirmed by the comet test. The increase in DNA reduction current with increasing compound concentration up to 10 µM was relatively small, and clear changes were seen at the concentration of 20 µM and led to DNA destabilization at 30 µM. In the comet assay, the interaction of compound **2** with DNA did not lead to measurable DNA strand breaks. After performing a comet assay and polarography measurements, it could be concluded that compound **2** shows a slight genotoxic effect—expressed by binding to DNA (seen by polarography), with a lack of damage in the comet assay. It is possible that compound **2** can induce DNA damage, which was not detectable in the conditions of our conventional comet assay. Our previous investigations (not published) performed on 14 olivacine derivatives showed that all compounds, except compound **2**, generated a large amount of genotoxic damage that was statistically significant. Compound **2** strongly activated p53 [18], and showed strong antitumor activity for cells showing multidrug resistance (the results of this research will be published soon). Moreover, those unpublished investigations revealed that compound **2** was non-toxic in the tested concentration range in normal human dermal fibroblasts and showed low toxicity in mouse fibroblasts (3T3/Balb − IC_50_ = 67 µM). Therefore, we are confident that compound **2** does not cause significant genotoxic damage in our tested methods. This was the reason why we abandoned comet assay modifications.

The changes observed for ellipticine were visible already at a concentration of 1 µM, DNA destabilization was observed at a concentration of 2 µM, and complete destabilization was observed at a concentration of 4 µM. At the same concentrations of ellipticine, genotoxic damage seen in the comet assay was observed.

A common feature in both compound **2** and ellipticine was the destabilization of DNA, leading to structural damage. It should be emphasized that despite the different mechanisms of interaction of the compounds with DNA, the final effect at high concentrations was similar, and the differences were due to the interaction mechanism. This would not be apparent after performing only the comet assay without additional polarographic studies. The comet assay also showed no similarities in the effects of compound **2** and ellipticine.

The inhibition of topoisomerase II activity by ellipticine [4,5,6,52], olivacine, and their derivatives [12,35,36] is also an important mechanism of antitumor action of these compounds. To determine whether our four tested olivacine derivatives can interact with topo II, some comparative investigations were performed. Two compounds (**2** and **4**) were chosen for the investigations because of the different mechanisms of interaction with DNA found in the comet assay and polarography. As a model compound, etoposide, a topo II inhibitor, was chosen [52]. In the comet assay, a range of concentrations of etoposide for which the amount of genotoxic damage increased relatively little was determined. The range was 5–10 µM, where the saturation of the DNA-topo II complex by etoposide occurred. Higher concentrations caused a significant etoposide concentration-dependent increase in the amount of DNA damage in the comet assay, whereas lower concentrations of etoposide were too low to block the DNA-topo II complex. It is significant that regardless of the test sample assembly sequence and the order in which etoposide and the test compounds were added to CCRF/CEM cell cultures, the amount of genotoxic damage in the comet assay was lower than the arithmetic sum of the results for etoposide (ETP) observed in independent cultures of CCRF/CEM cells.

The administration of compounds **2** and **4** blocked the formation of damage caused by the subsequent addition of ETP. When ETP was administered to the cultured cells before the tested compounds, only a slight increase in the amount of damage (up to 10%) was observed. The results allow us to conclude that regardless of the effect on DNA, the tested derivatives competed for the same binding sites to DNA-topo II complex as ETP. This confirms that one of the main mechanisms of action of the tested compounds was the inhibition of topoisomerase II activity, which in the case of the other tested pyridocarbazoles has been reported in the literature [4,5,6,35].

Moreover, the computational studies may suggest that all compounds could compete with etoposide (high binding affinity) and are able to bind to the binding site of topoisomerase. In addition, they may interact with DNA. The manner of binding to the DNA strongly depends on the structure of the inhibitor.

Etoposide is a model inhibitory topoisomerase II compound. Unfortunately, besides its inhibitory effect, it can also cause DNA damage [43]. Our results of molecular modeling revealed that compound **2** is the most potent topoisomerase inhibitor with the highest free energy (ΔG_binding_ = −53 kJ/mol). On the other hand, in compound **2**, the smallest genotoxic damage in the structure of DNA was observed. These properties of compound **2** make it very eligible for application in cancer therapy. Some degree of DNA damage is desirable because the cell is diverted to the apoptotic pathway. Previous papers of Jasztold-Howorko et al. [15] and Gębarowski et al. [18] reported an apoptotic effect of compound **2**. Additionally, compound **2** caused the largest increase in p53 and p21 protein levels of all the pyridocarbazoles tested [18]. As shown in this study using the comet assay, compound **2** is effective and has anticancer activity, but does not cause greater genotoxic damage in DNA. Modifications of compounds cause changes in affinity to topoisomerase II. Compound **4** was a weaker inhibitor of topoisomerase II considering the estimated free energy of binding (ΔG_binding_ = −47.5 kJ/mol). Compound **4** in a concentration of 10 µM caused the most genotoxic damage of all the compounds tested. On one hand, this may be desirable, while on the other hand, it may cause other cancers to form in the body. Compound **3** is the smallest of all the compounds tested because it has the shortest aliphatic chain and has the lowest free energy of binding (ΔG_binding_ = −42 kJ/mol). In addition, compound **3** exhibited strong genotoxic effects similar to compound **4**. For this reason, it appears to be the least beneficial topoisomerase II inhibitor. Compound **1** showed similar affinity for topoisomerase as compound **4** (ΔG_binding_ = −47.9 kJ/mol). Moreover, compound **1** caused severe genotoxic damage, especially at a concentration of 10 µM. Due to additional unfavorable donor–donor and acceptor–acceptor interactions, it is not a good candidate for an anticancer drug.

To extend our knowledge about the action of olivacine derivatives with DNA, the histone acetylation was examined. Unfortunately, no influence on histone acetylation was observed. The decrease in acetylated histones was proportional to the vitality decrease, observed in the MTT test.

According to the literature, pyridocarbazoles increase the level of p53 protein in cancer cells [53]. Our previous studies have demonstrated the ability of the tested pyridocarbazoles to reactivate selected anticancer functions of the p53 protein in mutant p53-containing cells (CCRF/CEM). In previous studies [15,16,18], we also used other cell lines, both normal and cancer. Our previous studies did not reveal a cytotoxic effect on normal cell line BALB/3T3 [15]. The tested pyridocarbazoles interact with DNA and activate the intracellular damage recognition kinase pathways, which should also result in the phosphorylation and activation of p53 protein observed in previous studies [18]. Increased stability and activity of p53 lead to the inhibition of topo II transcription and a decreased rate of DNA replication in cancer cells [50]. In this study, the direct interactions of p53 protein and topo II were not investigated. Instead, we focused on evaluating the direct interactions of the tested pyridocarbazoles with the topo II/DNA complex and demonstrating the competition between the tested compounds and the standard topo II inhibitor (etoposide) for access to topoisomerase. However, by stabilizing cleavable DNA complexes, the studied pyridocarbazoles activate intracellular damage recognition kinase pathways, which should also result in phosphorylation and activation of p53 protein.

Compounds **2** and **4** should be examined more, both in vitro and in vivo, using an experimental animal model, because of the promising profile of anticancer activity and the prospect of future use in cancer therapy. The research presented in this article has shown that even minor modifications of compounds cause significant changes in their properties. The type of modification presented in compound **2** is the most beneficial, as this compound has the weakest genotoxic effect and the highest affinity to topoisomerase II.

## 4. Materials and Methods

### 4.1. Tested Compounds

Four new olivacine derivatives, compounds **1**–**4** (Figure 2) as well as ellipticine (Figure 1a) were tested in this work. Compounds **1**–**4** were synthesized in the Department of Organic Chemistry of Wroclaw Medical University. The control substance, synthetic ellipticine (CAS: 519-23-3), was bought from Sigma-Aldrich (Saint Louis, MO, USA). The 3 mM stock solutions of all tested compounds and ellipticine were prepared in DMSO and were stored at −80 °C.

### 4.2. Cell Line

A suspension of the acute human lymphoblastic leukemia cell line CCRF/CEM was used in most of the experiments. For histone acetylation, the MCF-7 cell line was used. Both lines were obtained from the collection of The European Collection of Authenticated Cell Cultures. The cells were cultivated in a CO_2_ incubator at 37 °C and with 5% CO_2_. The culture medium for the CCRF/CEM cell line was RPMI-1640 (Sigma-Aldrich). MCF-7 cells were grown in EMEM medium (Sigma-Aldrich). All media cultures were supplemented with the addition of 10% FBS (fetal bovine serum), other supplements, and antibiotics (1% glutamine, streptomycin, penicillin).

### 4.3. Cell Viability

The impact of the compounds on viability of the CCRF/CEM cell line was evaluated. Cell were treated with the tested compounds for 2 h. Cells were collected into centrifuge tubes and were then centrifuged at 600× *g* for 5 min. Supernatant was removed and the cells were suspended in PBS and propidium iodide was added to them. After 5 min of incubation in the dark, the samples were analyzed in the image-based cytometer Arthur (NanoEnTek Inc., Waltham, MA, USA).

The 3-(4,5-dimethylthiazol-2-yl)-2,5-diphenyltetrazolium bromide (MTT) assay was used to evaluate the effect of tested compounds on cell vitality according to the ISO 10,993 standard, part V. CCRF/CEM (2 × 10^5^ cell per well) and MCF-7 (2 × 10^4^ cell per well) cell lines were seeded onto 96-well plates. Twenty-four hours after seeding, cells were exposed to different concentrations of the tested compounds for 24 h incubation. After incubation, the medium was removed and 50 μL of the MTT solution (1 mg/mL) was added and then incubated for 2 h at 37 °C. Finally, colored formazan was dissolved in isopropanol. The percentage of viable cells was calculated by measuring the absorbance of the colored formazan reaction product at 570 nm using a Varioskan LUX Multimode Microplate Reader (Thermo Fisher Scientific, Waltham, MA, USA).

### 4.4. Comet Assay—Single Cell Gel Electrophoresis under Alkaline Conditions

Single cell gel electrophoresis (SCGE), also known as the comet assay, was performed in our laboratory according to our own procedure, based on the literature [29,40,54]. It was performed using CCRF/CEM cell lines. CCRF/CEM cells were washed twice before the test in buffered saline solution (PBS) without Ca^2+^ and Mg^2+^ ions and then suspended in the amount of 5 × 10^5^/mL in the same PBS. The compounds were added to the cells prepared in this way and incubated with them for 2 h in the dark at 4 °C.

Immediately after the incubation, the cells were washed twice with buffered saline solution (PBS). The cells prepared in this way (in glass tubes, in a water bath at 37 °C) were suspended in PBS and an equal volume of 1% low-temperature gelification agarose (LMP agarose) was added to them. The cell and agarose suspension was applied on the microscopic slides placed on a warm plate (37 °C) and covered with cover slips. The specimens prepared in this way were placed on a metal block and cooled to 4 °C to quickly clot the agarose. Next, the specimens were placed in the refrigerator for about 30 min. The microscopic slides were prepared before the experiment and were covered with a 0.5% solution of normal melting point agarose (melting point = 65 °C).

After clotting of the agarose, the cover slips were removed and the specimens were placed in cold (4 °C) lysis buffer (pH 10.0) that contained 2.5 M NaCl, 1% Triton X-100, and 100 mM Na_2_EDTA, 10 mM Tris, and 10% DMSO. The specimens were left in lysis buffer for about 18 h in the dark (4 °C) until the next day. Further processing took place in the dark in a cold room (4 °C).

After the lysis, the lysis buffer was rinsed from the specimens (four times, 5 min each) in electrophoresis buffer (300 mM NaOH, 1 mM Na_2_EDTA; pH 13.0) and then placed in a horizontal gel electrophoresis system in electrophoresis buffer. The specimens were left in the electrophoresis system for 45 min. During this time, alkaline denaturation took place, which made it possible to reveal breaks in the strands and alkali-stable sites. Next, cellular DNA electrophoresis was performed under the conditions recommended for the test: 1.2 V, 300 mA, for 20 min.

After the electrophoresis, the specimens were rinsed (four times, 3 min each) with cold neutralizing buffer (Tris 0.4 M, pH = 7.5) and then stained with fluorescent dye 4’-6-diamidine-2-phenylindol (DAPI, 1 µg/mL in aqueous solution) and covered with cover slips.

After coloring, the specimens were left in the dark, in an airtight, moist chamber at 4 °C. The analysis of the preparations was carried out the next day after staining and electrophoresis.

The analysis of comet specimens was carried out using the Eclipse E-600 fluorescent microscope (Nikon, Japan) equipped with Plan Fluor lenses and UV 1A filter block. The acquisition of comet images was performed using a 1 MP FireWire monochrome digital camera and CometPlus 2.5 from Theta System Electronics GmbH on a computer running Windows XP Professional.

Comet images were also analyzed using CometPlus 2.5 from Theta System Electronics GmbH. The analysis was carried out in real time and the results of the analyzed comet images were saved in the program’s database. The program used enables the evaluation of many parameters of the assessed comets. The following parameters were analyzed:

- the DNA content in the tail of the comet (%), and
- the length of the comet tail.

### 4.5. Polarography

Polarographic measurements were performed using an MTM M161 electrochemical analyzer (MTM Anko, Krakow, Poland) with the M164C cell stand (MTM Anko, Krakow, Poland) in a three-electrode cell configuration. A HMDE (1.3 mm^2^) was the working electrode, an Ag/AgCl/KCl 3M was the reference electrode, and a platinum wire served as the auxiliary electrode. Experiments were carried out under an argon atmosphere at 25 °C in 0.1 mol/dm^3^ acetate buffer, pH 5.6, as the medium. The solutions were purged with argon (Ar-99%) for 10 min before each experiment. The differential pulse polarographic (DPP) measurements on the HMDE were performed with a pulse amplitude of −25 mV, a scan range from −0.8 V to −1.5 V, and at a scan rate of 5 mV/s. Before each measurement, the solution was conditioned at −0.1 V for 30 s without mixing.

DNA was dissolved in 0.1 M acetate buffer by mixing it for 48 h in the dark, at 40 °C. The required volume of stock solution of the tested compounds, dissolved previously in DMSO, was taken before each measurement and diluted in 0.1 mol/dm^3^ acetate buffer to achieve the following concentrations: 25 µg/mL for DNA, 0.2% for DMSO, 1, 5, 10, 20, 30 µM for the tested compounds, and 1, 2, 3, 4 µM for ellipticine.

DNA concentration was first defined spectrophotometrically using a CECIL CE 3021 spectrophotometer (Cecil Instruments Ltd.) at 25 °C on the day of the experiment. Added 0.2% DMSO did not affect the polarographic curves.

### 4.6. Histone Acetylation

The histone acetylation was performed using a CycLex Cellular Histone Acetylation Assay Kit [55]. This kit is designed for chromogenic detection (ELISA) of relative levels of acetylated histones in MCF-7 cell cultures, grown in 96-well microplates. Before the measurements, all reagents were brought to room temperature. All reagents in the kit were ready to use.

The plates with the cells were incubated at 37 °C overnight in a CO_2_ incubator. Then, appropriate amounts of test compounds were added to each well and the microplates were incubated at 37 °C for an appropriate time (from 2 to 360 min). After applying the single and dual fixation protocols, and adding the antibodies and reagents, the absorbance was measured at dual wavelengths of 450/540 nm.

### 4.7. Molecular Modeling

All the tested compounds were optimized at the PM6 level of theory taking into account the solvent effect (polarizable continuum model, PCM) by using the Gaussian 09 package (Gaussian Headquarters, Wallingford, CT, USA) [56,57,58]. To predict the binding mode of compounds into the binding site of topoisomerase, the AutoDock 4.2 package (The Scripps Research Institute, La Jolla, CA, USA) with a standard protocol was used [59]. As an input, we used a specially prepared crystal structure of topo IIα (PDB ID 5GWK) downloaded from the Protein Data Bank (PDB) (https://www.rcsb.org/structure/5gwk, accession on 18 June 2021) [60]. Validation of the docking procedure was performed by docking etoposide into the crystal structures of topoisomerase and comparing its position in the original crystallographic structure. The root mean square deviation (RMSD) was calculated to measure the docking prediction accuracy on the LigRMSD web server [61]. The pose was optimal when its RMSD was found to be less than 1.5 Å. The protein and ligand preparation and docking procedure were described in detail in previous studies [62,63]. The obtained results were visualized using a Chimera and a BIOVIA Discovery Studio visualizer (Dassault Systèmes Corporate, Dassault Systèmas, Waltham, MA, USA) [64].

### 4.8. Statistical Analysis

All results presented here are E/E_0_ ratios given as the mean ± SEM (standard error of the mean), where E is the result for the culture with the addition of the tested pyridocarbazoles and E_0_ is a control without the tested compound. The statistical analysis was performed using GraphPad Prism 7 Software (GraphPad Software, San Diego, CA, USA). The data were tested to check the normal distribution by the Shapiro–Wilk test. The unpaired *t*-test was applied for parametric data. A value of *p* < 0.05 was considered statistically significant.

## 5. Conclusions

The present article describes a series of investigations on four olivacine derivatives. Several previous studies have demonstrated the anticancer activity of the tested compounds and their influence on p53 protein level. In this study, we extended the spectrum of investigations to comet assay, polarography, inhibition of topoisomerase II activity, and molecular docking studies of the tested compounds.

The comet assay is widely used by scientists in toxicology and during the assessment of genotoxic effects of newly tested compounds. However, as we have described, it does not always show all the effects of interactions with DNA. In turn, polarography allows one to look more widely than a comet assay and can reveal a different aspect of the investigations. Comparison of the two types of test indicates that compounds that do not cause DNA damage visible in the comet assay can directly interact with DNA, which was visible in the polarography measurements.

Comparing the results of the comet assay and the polarography, we can see that the damage visible in the comet assay is the result of the destabilization of the DNA chains. Polarographic measurements indicate that the interaction of the tested compounds with DNA first increases the stability of the nucleic acid structure. The increase in compound concentrations leads to the occurrence of damage, which results in destabilization of the DNA structure. This is proof that these compounds have antitumor action.

Polarography seems to be a good method for preliminary screening tests of new drugs due to the speed and simplicity of performance in the electrochemistry laboratory. It enables one to observe the interaction of compounds with DNA, invisible in the comet assay including reduction, destabilization, and intercalation. Polarography allows one to look at the action of the compound from the perspective of the mechanism of action, while the comet assay can only show the effect. In polarography, we can also qualitatively assess the genotoxic effects of the tested substances and determine the concentration that causes DNA destabilization. In this respect, all the new tested derivatives present much weaker effects than ellipticine, but they still have the potential to become antitumor agents.

All the tested compounds can have an impact on DNA, as reported in this paper. They can also bind to topoisomerase II, which was shown by molecular docking studies. Changes in the structure of the compounds affect the strength of interaction with this enzyme. The best topoisomerase II inhibitor was compound **2**, and it caused the least genotoxic damage. On the other hand, compound **4** had a strong genotoxic effect but its affinity to topoisomerase II was quite weak.

Oncologic therapy should focus in future on small targeted molecule therapies such as DNA-targeting drugs. Thus, it is very important to identify such drugs, and pyridocarbazole derivatives are good candidates, especially compound **2**.

## Figures and Tables

**Table 1:** Electrochemical data of DNA reduction peak according to the compounds’ concentrations.

Concentration [µM]	E_p_ [mV]	I_p_ [µA]	I_p_/I_pDNA_
Compound **1**			
0	−1340	−0.2840	1.00
1	−1340	−0.2698	0.95
5	−1340	−0.2583	0.94
10	−1345	−0.2620	0.92
20	sh	sh	-
Compound **2**			
0	−1335	−0.2897	1.00
1	−1335	−0.2940	1.01
5	−1340	−0.3116	1.08
10	−1345	−0.3370	1.16
20	−1350 (sh)	−0.4440 (sh)	1.53
Compound **3**			
0	−1335	−0.3141	1.00
1	−1335	−0.3170	1.01
5	−1335	−0.3128	0.99
10	−1335	−0.2988	0.95
20	−1340	−0.2630	0.84
Compound **4**			
0	−1340	−0.3146	1.00
1	−1340	−0.3112	0.99
5	−1340	−0.2883	0.92
10	−1340	−0.2695	0.86
20	−1345	−0.2510	0.79
Ellipticine			
0	−1335	−0.2680	1.00
1	−1335	−0.2824	1.05
2	−1360	−0.3620	1.35
3	−1350	−0.4400	1.64
4	sh	sh	-

sh—shoulder on the curve.

**Table 2:** Evaluation of genotoxic effects of the tested compounds compared to ETP (for average ETP concentration from the range 5–10 µM, which was 7.5 µM). The result was expressed as the proportion to the ETP caused damage in three tests, where the reference value for etoposide is E_ETP_ = 3.

Pyridocarbazole Concentration	Compound 2	Compound 4
Compounds added before ETP		
5 μM	1.71	2.82
10 μM	1.70	2.26
20 μM	1.53	2.51
ETP added before compounds		
5 μM	3.19	3.32
10 μM	3.06	3.12
20 μM	3.18	3.23

**Table 3:** Evaluation of genotoxic effects of compounds in comparison with ∑ETP + pyridocarbazoles. The result was expressed as the proportion to the sum of ETP and pyridocarbazole caused damage made in three tests, where the reference value for the sum of etoposide and pyridocarbazoles was E_ETP + PYR_ = 3.

Concentration	Compound 2	Compound 4
Compounds added before ETP		
5 μM	1.42	1.60
10 μM	1.40	1.17
20 μM	1.23	1.26
ETP added before compounds		
5 μM	2.92	1.40
10 μM	2.72	1.37
20 μM	2.91	1.23

**Table 4:** Free energy of binding and intermolecular interactions of designed compounds with topoisomerase IIα in complex with DNA.

	Compound 1	Compound 2	Compound 3	Compound 4	Etoposide
**ΔG_binding_** **[kJ/mol]**	−7.9	−53.0	−42.2	−47.5	−44.6
**conventional HB**	Asp463 Leu486 DT9	Asp463 Leu486	DT9 DG10	Asp463 Leu468 DC8	Asp463 DG13
**carbon HB**	-	Arg487 Gly488	DT9	Arg487 DG13	Gly462 Asp463
**π–π stacking**	DC8 DC9 DC13	DC8 DC9 DG13	-	DC8 DT9 DG13	DT9 DT13
**alkyl** **π–alkyl**	Arg487 Met762	DC8 DT9	DC8 DG13	Met762 DC8 DA12	Arg487 Met762 Pro803
**π-σ**	-	DG13	DT9	DT9	-
**amide–π stacking**	-	-	Arg487	-	Arg487
**π–π T-shape**	-	-	DT9	-	-
**van der Waals**	Glu461 Gly462 Ser464 Gly760 DG10 DA12	Glu461 Gly462 Ser464 Pro485 Gly488 DG10	Glu461 Gly462 Ser464 Gly488 DC11 DA12	Gly462 Ser464 Gly488 DG10	Glu461 Gly488 Met766 DC8 DG10 DA12
